# Absolute values of regional ventilation-perfusion mismatch in patients with ARDS monitored by electrical impedance tomography and the role of dead space and shunt compensation

**DOI:** 10.1186/s13054-024-05033-8

**Published:** 2024-07-15

**Authors:** Marco Leali, Ines Marongiu, Elena Spinelli, Valentina Chiavieri, Joaquin Perez, Mauro Panigada, Giacomo Grasselli, Tommaso Mauri

**Affiliations:** 1 Department of Pathophysiology and Transplantation, University of Milan, Milan, Italy; 2 Department of Anesthesia, Critical Care and Emergency, Fondazione IRCCS Ca’ Granda Ospedale Maggiore Policlinico, Milan, Italy

**Keywords:** Ventilation/perfusion, V′/Q, Electrical impedance tomography, EIT, ARDS, Calibration, Non-invasive, Shunt, Dead space, Compensation

## Abstract

**Background:**

Assessment of regional ventilation/perfusion (V′/Q) mismatch using electrical impedance tomography (EIT) represents a promising advancement for personalized management of the acute respiratory distress syndrome (ARDS). However, accuracy is still hindered by the need for invasive monitoring to calibrate ventilation and perfusion. Here, we propose a non-invasive correction that uses only EIT data and characterized patients with more pronounced compensation of V′/Q mismatch.

**Methods:**

We enrolled twenty-one ARDS patients on controlled mechanical ventilation. Cardiac output was measured invasively, and ventilation and perfusion were assessed by EIT. Relative V′/Q maps by EIT were calibrated to absolute values using the minute ventilation to invasive cardiac output (MV/CO) ratio (V′/Q-ABS), left unadjusted (V′/Q-REL), or corrected by MV/CO ratio derived from EIT data (V′/Q-CORR). The ratio between ventilation to dependent regions and perfusion reaching shunted units (${\text{V}}_{{\text{D}}}^{\prime }$/Q_SHUNT_) was calculated as an index of more effective hypoxic pulmonary vasoconstriction. The ratio between perfusion to non-dependent regions and ventilation to dead space units (Q_ND_/${\text{V}}_{{{\text{DS}}}}^{\prime }$) was calculated as an index of hypocapnic pneumoconstriction.

**Results:**

Our calibration factor correlated with invasive MV/CO (r = 0.65, *p* < 0.001), showed good accuracy and no apparent bias. Compared to V′/Q-ABS, V′/Q-REL maps overestimated ventilation (*p* = 0.013) and perfusion (*p* = 0.002) to low V′/Q units and underestimated ventilation (*p* = 0.011) and perfusion (*p* = 0.008) to high V′/Q units. The heterogeneity of ventilation and perfusion reaching different V′/Q compartments was underestimated. V′/Q-CORR maps eliminated all these differences with V′/Q-ABS (*p* > 0.05). Higher $V_{D}^{\prime } /Q_{SHUNT}$ correlated with higher PaO_2_/FiO_2_ (r = 0.49, *p* = 0.025) and lower shunt fraction (ρ =  − 0.59, *p* = 0.005). Higher $Q_{ND} /V_{DS}^{\prime }$ correlated with lower PEEP (ρ =  − 0.62, *p* = 0.003) and plateau pressure (ρ =  − 0.59, *p* = 0.005). Lower values of both indexes were associated with less ventilator-free days (*p* = 0.05 and *p* = 0.03, respectively).

**Conclusions:**

Regional V′/Q maps calibrated with a non-invasive EIT-only method closely approximate the ones obtained with invasive monitoring. Higher efficiency of shunt compensation improves oxygenation while compensation of dead space is less needed at lower airway pressure. Patients with more effective compensation mechanisms could have better outcomes.

**Supplementary Information:**

The online version contains supplementary material available at 10.1186/s13054-024-05033-8.

## Background

Heterogenous distribution of pulmonary ventilation (V′) and perfusion (Q), with regions receiving excessive perfusion and other more ventilated than perfused, is a hallmark of the acute respiratory distress syndrome (ARDS) [1].

The V′/Q ratio ranges from 0 (shunt compartment) to infinity (dead space compartment) and the presence of lung units with V′/Q different from 1 (i.e., the ideal V′/Q match) alter gas exchange causing hypoxemia and/or hypercapnia, which are correlated with poor outcomes [2, 3]. Experimental studies [4–6] also showed that, in presence of large defects of regional ventilation or perfusion, multiple mechanisms induce bilateral diffuse lung injury in healthy animals. These include regional hypoperfusion, alveolar collapse, redistribution of ventilation from hypo-perfused areas, alveolar hypocapnia and inflammation. Of note, physiologic compensation of shunt and dead space plays a key role in the development of de novo lung injury induced by V′/Q mismatch. In summary, V′/Q mismatch recently emerged as a prognostic marker of the severity of ARDS and as a mechanism of lung injury.

In clinical practice, methods to precisely assess regional compartments of V′/Q mismatch are still lacking. Classic physiological measures such as shunt fraction calculated by the Berggren equation [7] and dead space obtained by volumetric capnography [8] are hindered by multiple limitations: they only consider one side of the ventilation/perfusion defects; they can’t assess regional distribution of V′/Q mismatch compartments; and they are highly dependent from physiologic confounders, such as hemodynamics and compensation mechanisms.

Electrical impedance tomography (EIT) is a radiation-free, non-invasive bedside lung imaging tool, able to dynamically assess the distribution of the regional fractions of ventilation and perfusion within the lungs [9, 10]. EIT allows precise quantification of the relative V′/Q ratio for each lung unit (i.e., the % of V′ divided by the % of Q reaching each pixel) with much less dependency from external confounders, due to its topographic rather than functional nature. In intubated patients, absolute values of ventilation distribution are easily obtained by calibration with the ventilator spirometer, while presence of invasive monitoring for cardiac output is needed to obtain regional perfusion and absolute values of pixel-level V′/Q mismatch. Otherwise, relative V′/Q mismatch obtained by sheer division of relative maps can lead to imprecise evaluations of the extent of regional V'/Q mismatch [11].

Previous studies showed that EIT can also measure a smaller pulsatility signal, which correlates with stroke volume [12, 13]. While it may not be as accurate as needed for hemodynamic monitoring in the critically ill, we thought that the precision of the pulsatility signal could be sufficient to calibrate relative V′/Q mismatch and obtain EIT-based absolute values, without need of invasive monitoring. Increased robustness and availability of EIT-only absolute V′/Q mismatch data could become a more solid and widespread guide for treatment of ARDS.

Here, we enrolled a convenience sample of representative intubated ARDS patients with clinical indication for invasive monitoring of cardiac output to derive a calibration method based only on EIT data and transform relative measure of V′/Q mismatch to absolute values. Moreover, we explored the correlation between physiological compensation of shunt and dead space and pathophysiological severity and clinical outcome of patients.

## Methods

### Patient population

We enrolled 21 patients admitted to the Intensive Care Unit (ICU) of the Ospedale Maggiore Policlinico (Milan, Italy) with diagnosis of ARDS [14]. Patients were intubated, sedated, paralysed and mechanically ventilated, a central venous line was in place and cardiac output was monitored by pulmonary artery thermodilution (Swan-Ganz Oxymetry Paceport TD Catheter and HemoSphere Advanced Monitor, Edwards Lifesciences), or by peripheral femoral artery thermodilution (PiCCO catheters, IntelliVue MX750 monitor, Philips). Exclusion criteria were age < 18; hemodynamic instability (i.e., severe hypotension with systolic arterial pressure < 60 mmHg despite vasoactive support; systolic arterial pressure > 180 mmHg; hemodynamically significant cardiac arrhythmias); pregnancy; contraindications to the use of EIT (e.g., presence of pacemaker or chest surgical wounds dressing).

The study was approved by the Ethical Committee of the Policlinico Hospital, Milan, Italy (reference number 311_2021) and informed consent was obtained according to local regulations.

### Study protocol

At enrolment, the following data were collected: demographic data, etiology, Simplified Acute Physiology Score II (SAPS II) at ICU admission, SOFA score, controlled mechanical ventilation settings and arterial blood gas analysis. Based on history, ARDS etiology was divided into pulmonary versus extrapulmonary [15] and infectious vs. non-infectious. Patients were supine, in semi-recumbent position and an EIT dedicated belt with 16 electrodes was placed around the patient’s chest at the fifth or sixth intercostal space and connected to an EIT monitor (PulmoVista® 500, Dräger, Lübeck, Germany). EIT data were acquired at a frame rate of 50 Hz.

Ventilator settings were left as selected by the attending physician throughout the whole study. At the beginning of the study, respiratory mechanics (driving and plateau pressure, tidal volume, and minute ventilation) and hemodynamic data (heart rate, systemic arterial and central venous pressure) were collected, and arterial and central venous blood gases were analyzed.

Cardiac output was measured by thermodilution either via the pulmonary artery catheter, or by peripheral arterial thermodilution [16], as the mean of three replicates. Then, lung perfusion was promptly assessed by first-pass kinetics of a hypertonic saline bolus [17]. Perfusion assessment was as close as possible to cardiac output measurements (i.e. within 2–3 min): a 60 s baseline recording, free of any hypertonic saline interference, was acquired for ventilation and dynamic pulsatility analysis; then, an end-inspiratory breath hold lasting 20 s was performed. Two seconds after start of the occlusion, a bolus of 10 ml of 5% NaCl was rapidly injected via the central venous catheter.

### Physiological data

Shunt fraction was calculated using the Berggren equation [1], with a central venous sample used as a surrogate for mixed venous gases when a pulmonary arterial catheter was not in place. The ventilatory ratio was calculated as previously described [18], as a bedside index related to dead space.

### EIT data

Functional EIT (fEIT) images.

EIT images were reconstructed via Draeger’s proprietary algorithm, which is based on a finite element model (FEM)-based Newton Raphson algorithm [9], to 32 × 32 pixel matrices. Custom analyses were then performed with MatLab R2022a. Three types of functional EIT images (fEIT) were extracted:

- *ventilation maps* (V′), as the pixel-by-pixel change in impedance between inspiration and expiration ∆Z_TIDAL,PX_, averaged over five consecutive breaths [9].
- *pulsatility maps*, as the pixel-by-pixel change in impedance due to the cardiac-related signal (∆Z_CR,PX_), a smaller periodic signal superimposed to ventilation [19]. The cardiac-related signal was extracted dynamically using a modified version of a previously published algorithm (see reference [20] and Supplement).
- *lung perfusion maps* (Q), derived from the first-pass kinetics of the hypertonic saline bolus, as previously described [21].

Ventilation and perfusion to dependent and non-dependent units were calculated by splitting the 32 × 32 EIT images into two equal halves comprising 16 × 32 pixels. Further details about signal processing can be found in the Supplement.

Calibration factor from EIT data to replace cardiac output measurements.

Pulsatility signal by EIT is known to be correlated with stroke volume (SV) [12, 13], so we hypothesized that it could be used to adjust ventilation and perfusion maps for the MV/CO ratio and, ultimately, to generate absolute regional V′/Q maps with EIT data only. A cardiac ROI was calculated based on the phase of the pulsatility signal and subtracted from the lung ROI, determined from the ventilation signal. We then summed the pulsatility signal of all pixels within the lung ROI, thus obtaining a cardiac-related impedance change $\Delta Z_{CR}$ of pulmonary pixels (see Supplement for further details). It was then postulated that by adjusting for heart rate (HR) and respiratory rate (RR) a quantity proportional to the MV/CO ratio could be obtained, from which a calibration factor (K_C_) could be derived by linear regression:

1 $$ \frac{MV}{{CO}} \approx \beta_{1} *\frac{{\Delta Z_{TIDAL} *RR}}{{\Delta Z_{CR} *HR}} + \beta_{0} = K_{C} $$

where β_0_ is the intercept and β_1_ the angular coefficient. V′/Q maps were then obtained by pixel-by-pixel division of the ventilation (V′) and perfusion (Q) maps after thresholding: pixels above the 10% of maximum ventilation/perfusion within the image were considered as ventilated/perfused, respectively [22]. Three types of V′/Q maps were calculated:

- “relative” (V′/Q-REL), by mere division of the % of ventilation and perfusion reaching each pixel:
  2 $$ V^{\prime } /Q_{REL} \left( {i,j} \right) = \frac{{V^{\prime } \left( {i,j} \right)}}{{Q\left( {i,j} \right)}} $$
- “absolute” (V′/Q-ABS), by multiplication with minute ventilation (MV) and cardiac output (CO):
  3 $$ V^{\prime } /Q_{ABS} \left( {i,j} \right) = \frac{MV}{{CO}} \cdot \frac{{V^{\prime } \left( {i,j} \right)}}{{Q\left( {i,j} \right)}} $$
- “corrected” (V′/Q-CORR), by adjusting the V′/Q-REL values with the EIT-based novel calibration factor, without need of using invasive CO monitoring:
  4 $$ V^{\prime } /Q_{CORR} \left( {i,j} \right) = K_{C} \cdot \frac{{V^{\prime}\left( {i,j} \right)}}{{Q\left( {i,j} \right)}} = K_{C} \cdot V^{\prime } /Q_{REL} \left( {i,j} \right) $$

The percentage of ventilation and perfusion to 5-compartment (shunt, low V′/Q, normal V′/Q, high V′/Q, dead space) and 21-compartment (V′/Q decimal logarithm from − 1 to 1 with intervals of 0.1) models were calculated, as previously described [22]. From the 21-compartment distributions, the mean V′/Q of ventilation ($\overline{{V^{\prime } }}$) and perfusion ($\overline{Q}$) and indices of V′/Q heterogeneity (logSD_V′_ and logSD_Q_ respectively) were calculated, in analogy with the multiple inert gas elimination technique (MIGET) [23].

Physiological compensation of shunt and dead space measured by EIT.

Two indices were calculated to assess the physiological compensation of shunt and dead space by EIT:

- $\frac{{V_{D}^{\prime } }}{{Q_{SHUNT} }}$ as the ratio between ventilation to dependent regions (%) and the percentage of perfusion reaching shunted lung units (i.e., those with V′/Q < 0.1). This index is lower when dependent collapse decreases regional ventilation (low ${\text{V}}_{{\text{D}}}^{\prime }$) and perfusion is not redistributed to aerated ventilated units (high Q_SHUNT_). Higher values reflect either less severe disease, or greater efficiency of hypoxic pulmonary vasoconstriction [24].
- $\frac{{Q_{ND} }}{{V_{DS}^{\prime } }}$ as the ratio between perfusion to non-dependent regions (%) and the percentage of ventilation to dead space units (V′/Q > 10). This index is lower when non-dependent overdistension decreases regional perfusion (low Q_ND_) and tidal volume is not redistributed to normally perfused regions (high ${\text{V}}_{{{\text{DS}}}}^{\prime }$). Higher values reflect either less severe disease, or greater efficiency of hypocapnic pneumoconstriction [1].

A schematic about these indices can be found in the Supplement (Fig. S1).

Outcomes.

After the end of the protocol, data on outcomes were collected, including: hospital mortality, hospital and ICU length of stay (LoS), ventilator-free days (VFDs) censored at day 28 [25].

Primary endpoint.

The study primary endpoint was to confirm differences between V′/Q-REL and V′/Q-ABS, and to describe, instead, similarity between the novel V′/Q-CORR method and V′/Q-ABS. While the good correlation between EIT data and regional ventilation is established [9], the limiting factor for estimating the MV/CO ratio is EIT-based stroke volume (SV). Previous studies reported a coefficient of correlation of 0.69 between pulsatility EIT signal and SV in conditions similar to ours [12]. Given the preliminary nature of this work, we aimed at reproducing this result with high power (0.95), as a condition for further analyses. The required sample size with a 95% confidence level was 21.

### Statistical analysis

Comparison within patients of the different methods to obtain EIT-based V′/Q maps was first performed by repeated measures ANOVA, or Friedman test, as appropriate. Bonferroni correction was applied to adjust for multiple comparisons between different V′/Q compartments (eight, i.e. four for ventilation and four for perfusion). Bonferroni post-hoc tests were performed: either t-test for paired data, or Wilcoxon signed rank test, as appropriate. The fEIT maps adjusted with invasive measures were used as a reference for multiple comparisons. Correlation between continuous variables was performed either with Pearson’s or with Spearman’s correlation coefficients, as appropriate. Ordinary least squares were used to estimate linear regression model parameters. Normality was tested with the Lilliefors test, sphericity with Mauchly’s test. A Bland–Altman plot [26] was used to visually assess the calibration factor against the measured MV/CO ratio. Normally distributed data are indicated as mean ± standard deviation, while median (interquartile range) is used to report variables significantly deviating from normality. A 0.05 confidence level was used to consider results significant. Statistical analyses were performed with MatLab R2022a.

## Results

### Patient sample

Twenty-one patients with ARDS were enrolled, of 61 ± 10 years old. The study population is described in Table 1. Mean SAPS II score at admission was 41 [37–46], and SOFA was 9 ± 3. PaO_2_/FiO_2_ was 180 ± 68 mmHg, 9 patients (43%) had mild ARDS, 10 (48%) moderate and 2 (9%) severe. Etiology was mainly pulmonary (76%) over extra-pulmonary, and infectious (57%). Minute ventilation (MV) and cardiac output (CO) were 8.3 ± 3.1 and 6.2 ± 1.6 l/min, respectively. The mean ratio between MV and CO (MV/CO) was 1.35 ± 0.46, and 15 patients (71%) had a MV/CO ratio above one.

**Table 1:** Main characteristics of the study population

ARDS patients (n = 21)	
*Demographics, etiology and outcomes*	
Age, years	61 ± 10
Female sex, no. (%)	10 (48%)
SAPSII at admission	41 (37–46)
Pulmonary ARDS, no. (%)	16 (76%)
Infectious ARDS, no. (%)	12 (57%)
SOFA score	9 ± 3
Hospital mortality, no. (%)	4 (19%)
ICU length of stay, days	12 (7–22)
Ventilator-free days (VFD) at day 28	21 (7–25)
*Physiological data*	
MV, l/min	8.25 ± 3.06
RR, bpm	16.05 ± 4.42
PEEP, cmH_2_O	10 (8–14)
Vt, ml/kg PBW	8.06 ± 1.09
Driving pressure, cmH_2_O	10.48 ± 2.40
PaO_2_/FiO_2_, mmHg	180 ± 68
Ventilatory ratio	1.47 ± 0.46
CO, l/min	6.22 ± 1.56
HR, bpm	89 ± 18
MAP, mmHg	74 (70–78)
MV/CO	1.35 ± 0.46

ARDS, Acute respiratory distress syndrome; CO, Cardiac output; HR, Heart rate; ICU, Intensive care unit; MAP, Mean arterial pressure; MV, Minute volume; PaO_2_/FiO_2_, Ratio between arterial oxygen tension and inspired oxygen fraction; PBW, Predicted body weight; PEEP, Positive end expiratory pressure; RR, Respiratory rate; SOFA, Sequential organ failure assessment; SAPSII, Simplified acute physiology score II; VFD, Ventilator-free days; Vt, Tidal volume

### Calibration factor

**Fig. 1:**
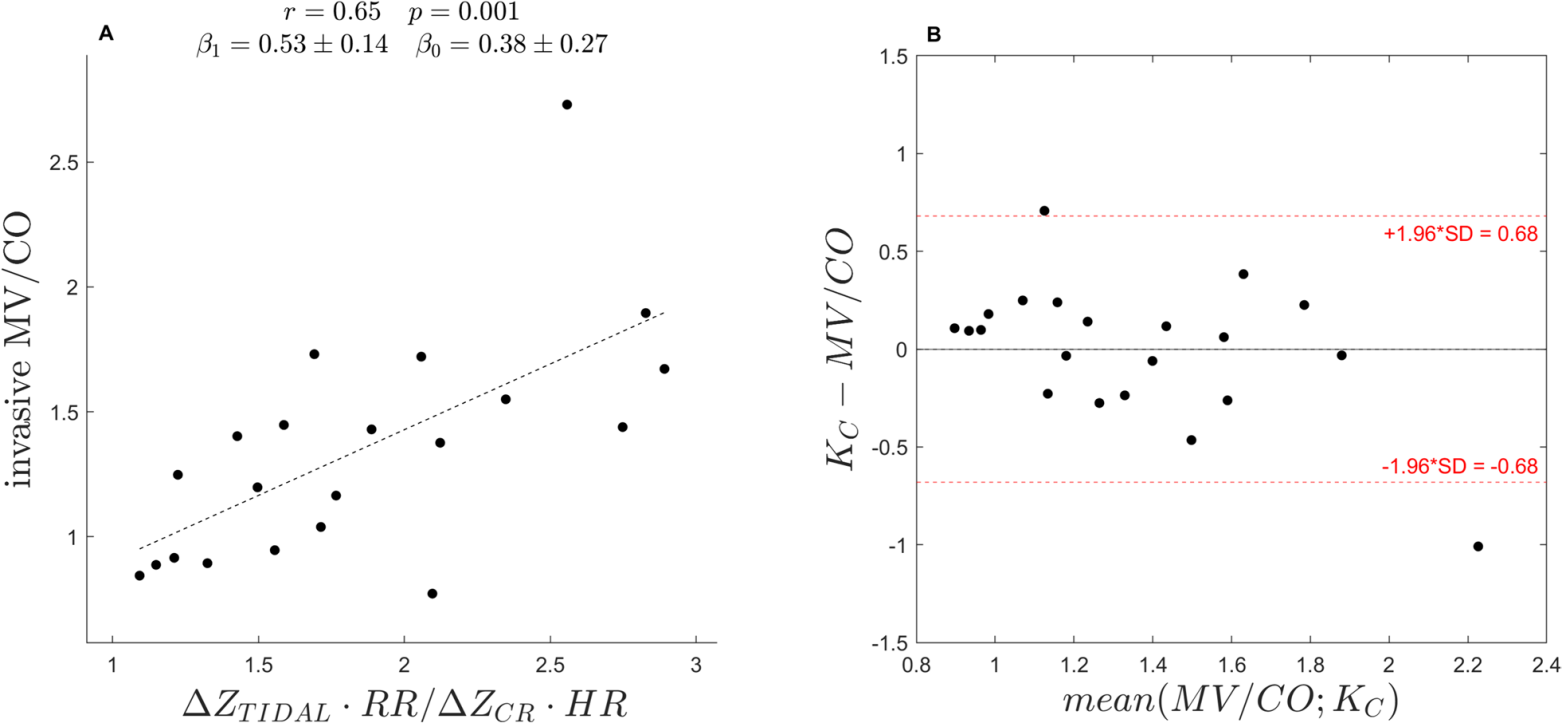
**A** The ratio between impedance changes due to tidal ventilation and cardiac pulsatility, when adjusted for heart and respiratory rate, correlates with the ratio between minute ventilation and cardiac output (MV/CO). **B** The Bland–Altman plot for the calibration factor (K_C_) calculated from the linear regression model in (**A**) and the MV/CO ratio. β_0,1_, Intercept and angular coefficient, respectively, of the linear regression model; CO, Cardiac output; HR, Heart rate; K_C_, Calibration factor; MV, Minute ventilation; RR, Respiratory rate; ∆Z_CR_, Cardiac-related impedance change; ∆Z_TIDAL_, Tidal change in impedance

We found a positive correlation coefficient of r = 0.65 (*p* < 0.001) between the MV/CO ratio measured invasively and the MV/CO ratio obtained by EIT only. A linear regression model yielded an angular coefficient β_1_ of 0.53 ± 0.14 and an intercept β_0_ of 0.38 ± 0.27 (Fig. 1A). A calibration factor for each patient was then derived from the model by substituting β estimates into Eq. (1), and the following average calibration factor was generated:

$$ K_{C} \approx 0.5*\frac{{\Delta Z_{TIDAL} *RR}}{{\Delta Z_{CR} *HR}} + 0.4. $$

The Bland–Altman plot showed no evidence for a fixed or proportional bias between the invasive MV/CO measures and the calibration factor (Fig. 1B). There was no evidence of the relationship between the calibration factor and MV/CO being influenced by PEEP (see Supplement). As expected, the relationship between the calibration factor and CO did not meet the criteria for CO monitoring per se (see Supplement and Fig. S2).

### Comparison between relative, absolute and corrected V′/Q values

**Fig. 2:**
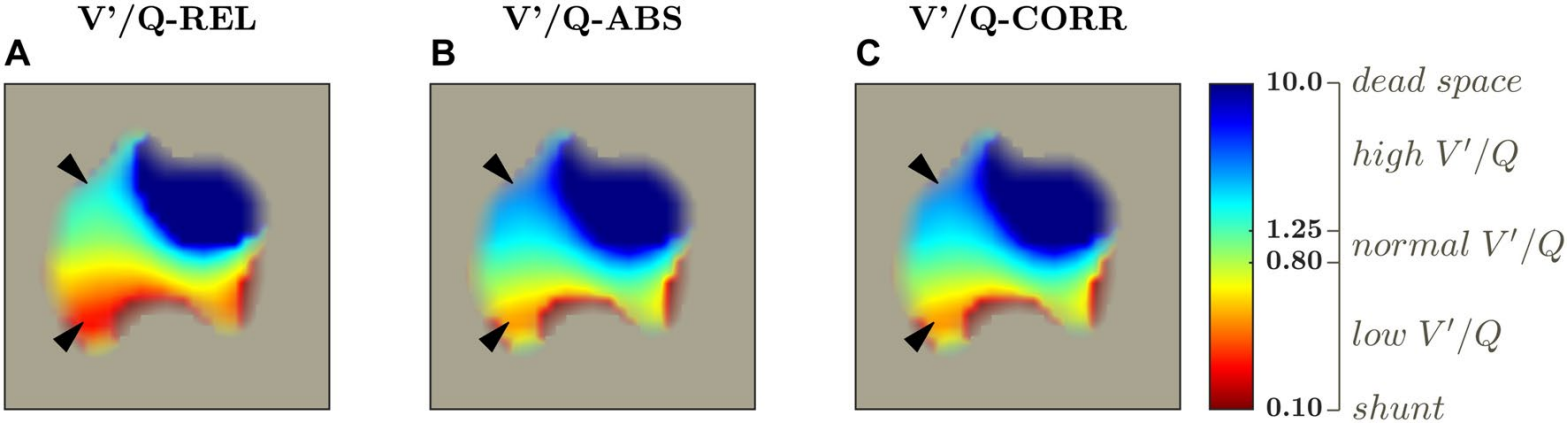
Representative V′/Q maps by mere superimposition without any correction (V′/Q-REL, *left column*), after correcting with invasive minute volume and cardiac output measurements (V′/Q-ABS, middle column) and after correcting with our proposed calibration factor (V′/Q-CORR, *right column*). This representative patient had a ratio between minute ventilation and cardiac output higher than one (MV/CO = 1.9). Note that without any calibration V′/Q is underestimated, with wider low V′/Q areas (*bottom arrowhead*) and narrower high V′/Q areas (*top arrowhead*), while after correcting with our proposed method this bias is not evident any more. The V′/Q ratio is expressed in base 10 logarithmic scale. V′/Q compartments [22] are shown in the colorbar

Figure 2 shows representative images for the three V′/Q maps that were generated for each patient, with arrows indicating regions with large differences between V′/Q-REL and V′/Q-ABS values (Fig. 2A vs. B) that disappeared when corrected for the calibration factor K_C_ (V′/Q-CORR, Fig. 2C).

**Fig. 3:**
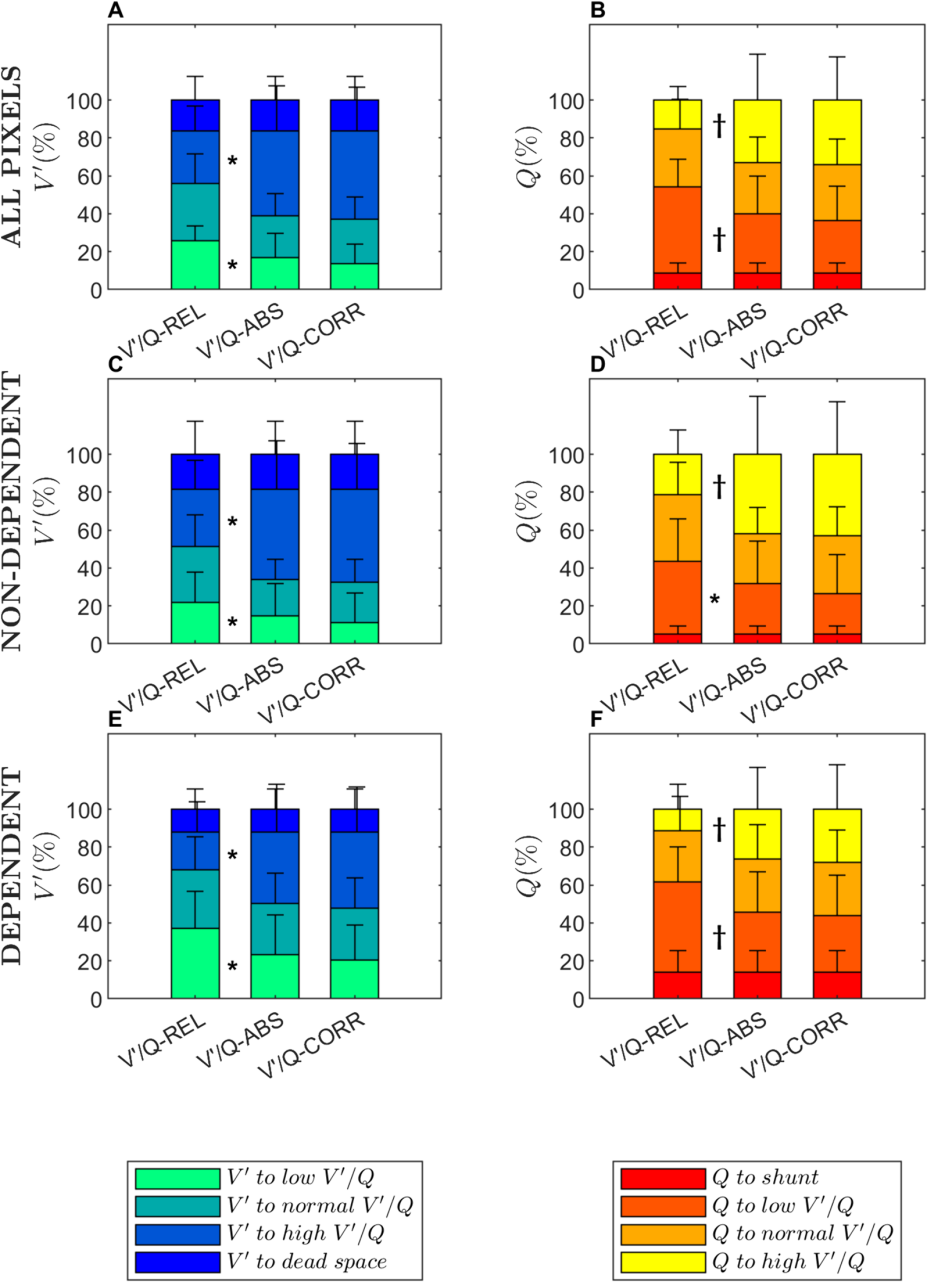
Percentage of ventilation (Left side panels) and perfusion (Right side panels) to V′/Q compartments of a 5-compartment model across the whole lungs (**A**, **B**) and in non-dependent (**C**, **D**) and dependent regions (**E**, **F**). Q, Perfusion; V′, Ventilation; V′/Q-ABS, “Absolute” V′/Q, i.e. adjusted with invasive measurements of minute volume and cardiac output; V′/Q-CORR, V′/Q corrected with our proposed calibration factor, i.e. with EIT data only; V′/Q-REL, “Relative” V′/Q, i.e. not calibrated. *p < 0.05; ^†^*p* < 0.01; ^☨^*p* < 0.001

When comparing 5-compartment data against the invasive reference (V′/Q-ABS), V′/Q-REL maps resulted in higher fraction of ventilation and perfusion to low V′/Q units (*p* = 0.013 and *p* = 0.002, respectively) and in lower percentages of ventilation and perfusion to high V′/Q units (*p* = 0.011 and *p* = 0.008, respectively). By our proposed correction method (V′/Q-CORR), these differences were no longer discernible (Fig. 3A, B).

Ventilation reaching dead space (V′/Q > 10) and perfusion reaching shunt (V′/Q < 0.1) compartments was almost identical across the three methods, as they depended mostly on non-ventilated and non-perfused pixels (for which relative and absolute methods perfectly correspond).

The same results were found in V′/Q maps generated for non-dependent and dependent lung regions (Fig. 3C–F).

**Fig. 4:**
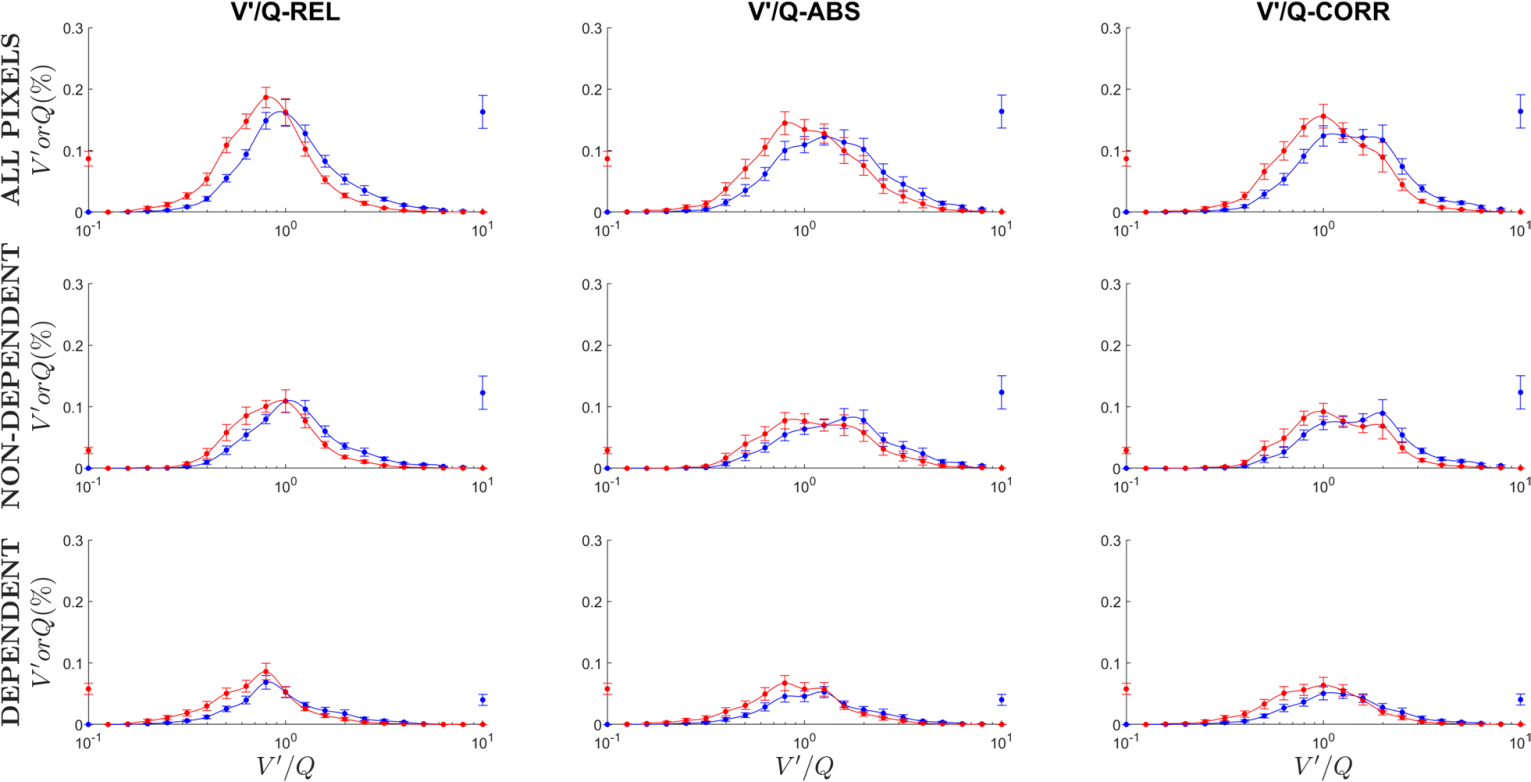
21-compartment distributions of ventilation (V′) and perfusion (Q) across different V′/Q ratios. Data is expressed as mean ± standard error across the entire population. Perfusion percentage is in red, ventilation percentage is in blue. Distributions have been derived from unadjusted V′/Q maps (V′/Q-REL, left row), V′/Q maps adjusted with invasive measurements of minute volume and cardiac output (V′/Q-ABS, middle row) and corrected with our proposed calibration factor (V′/Q-CORR). The top row displays distributions from all pixels, the middle row only from non-dependent units, the bottom row only from dependent units

Finally, 21-compartment distributions of ventilation and perfusion were obtained for each method to obtain V′/Q maps (Fig. 4). Both ventilation and perfusion from the invasive reference (V′/Q-ABS) showed a wide, bimodal shape, which was lost in the uncorrected (V′/Q-REL) maps. Our proposed correction resulted in a shape qualitatively much more similar, recovering bimodality. When comparing measures of mean V′/Q and heterogeneity of V′/Q mismatch (Table 2), V′-Q-REL map resulted in overestimation of mean V′/Q ratios and underestimation of V′/Q variability, which were no more present after correction.

**Table 2:** Descriptive metrics of 21-compartment model for V′/Q-REL, V′/Q-ABS and V′/Q-CORR maps (see “Methods” section)

	V′/Q-REL	V′/Q-ABS	V′/Q-CORR	*p* value
*All pixels*				
$\overline{{V^{\prime } }}$	1.56 ± 0.35**	1.96 ± 0.60	1.97 ± 0.46	0.001
logSD V′	0.40 ± 0.12**	0.37 ± 0.11	0.37 ± 0.11	< 0.001
$\overline{Q}$	0.69 (0.61–0.78)**	0.90 (0.61–1.09)	0.90 (0.70–1.01)	< 0.001
logSD_Q_	0.31 ± 0.07*	0.33 ± 0.08	0.34 ± 0.08	0.001
*Non-dependent*				
$\overline{{V^{\prime } }}$	1.84 ± 0.81**	2.28 ± 1.00	2.29 ± 0.93	0.001
logSD V′	0.36 ± 0.13**	0.33 ± 0.13	0.33 ± 0.12	< 0.001
$\overline{Q}$	0.83 ± 0.21**	1.14 ± 0.59	1.13 ± 0.47	0.001
logSD_Q_	0.26 ± 0.08*	0.28 ± 0.08	0.28 ± 0.08	0.005
*Dependent*				
$\overline{{V^{\prime } }}$	1.31 ± 0.68**	1.64 ± 0.76	1.66 ± 0.72	< 0.001
logSD V′	0.34 (0.30–0.42)**	0.33 (0.29–0.38)	0.31 (0.29–0.40)	0.001
$\overline{Q}$	0.60 ± 0.24**	0.75 ± 0.30	0.78 ± 0.34	< 0.001
logSD_Q_	0.32 ± 0.08*	0.35 ± 0.11	0.35 ± 0.10	0.001

$\overline{{V^{\prime } }}$ is the mean V′/Q of ventilation, $\overline{Q}$ is the mean V′/Q of perfusion, logSD V′ and logSD_Q_ are the logarithmic standard deviations of ventilation and perfusion distributions, respectively (see Methods). The metrics have been compared between all pixels, within non-dependent units and within dependent units. Significance of post-hoc tests is expressed as asterisks; tests have been performed with V′/Q-ABS as a reference group

*p < 0.05; **p < 0.01; ***p < 0.001

### Physiological variables correlated with shunt and dead space compensation

**Fig. 5:**
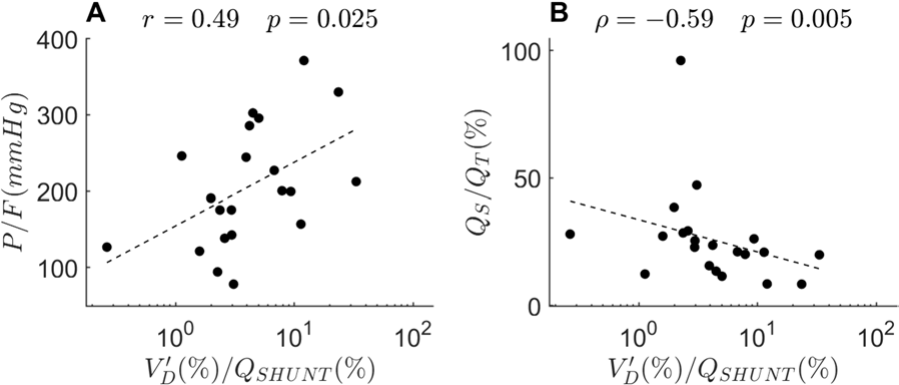
Shunt compensation and classical bedside measures of severity and shunt. See text for abbreviations. Indices from V′/Q maps corrected with our proposed calibration factor (V′/Q-CORR) are plotted against **A** P/F and **B** shunt fraction (Q_S_/Q_T_) from blood gases

The ratio between the fraction of ventilation to dependent regions and perfusion reaching shunt units ($V_{D}^{\prime } /Q_{SHUNT}$), an index of physiological compensation of shunt, correlated with the PaO_2_/FiO_2_ ratio (r = 0.49, *p* = 0.025) and, negatively, with the shunt fraction calculated from blood gases (ρ =  − 0.59, *p* = 0.005) (Fig. 5A, B). The correlations were almost identical across the three types of V′/Q maps, thus confirming the solidity of this index and independence for MV and CO (Fig. 3).

**Fig. 6:**
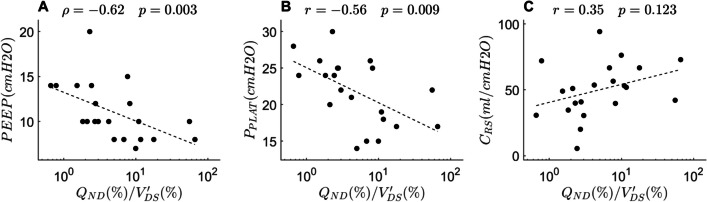
Dead space compensation and respiratory system mechanics. See text for abbreviations. Indices from V′/Q maps corrected with our proposed calibration factor (V′/Q-CORR, right column) are plotted against, **A** positive end expiratory pressure (PEEP), **B** plateau pressure (Pplat), and **C** respiratory system compliance (CRS)

Patients with a higher ratio between perfusion to non-dependent areas and ventilation to dead space units ($Q_{ND} /V_{DS}^{\prime }$), an index of more effective compensation of dead space, were ventilated with lower PEEP level (ρ =  − 0.62, *p* = 0.003) and had a lower plateau pressure (ρ =  − 0.59, *p* = 0.005), indicating less need for compensation at lower airway pressure, and showed a trend towards higher respiratory system compliance (r = 0.34, *p* = 0.126), indicating worsening regional compliance as method to redirect ventilation (Fig. 6A–C). Again, the relationships were nearly identical across the three types of V′/Q maps (Fig. S4).

### Shunt and dead space compensation is correlated with clinical outcomes

**Fig. 7:**
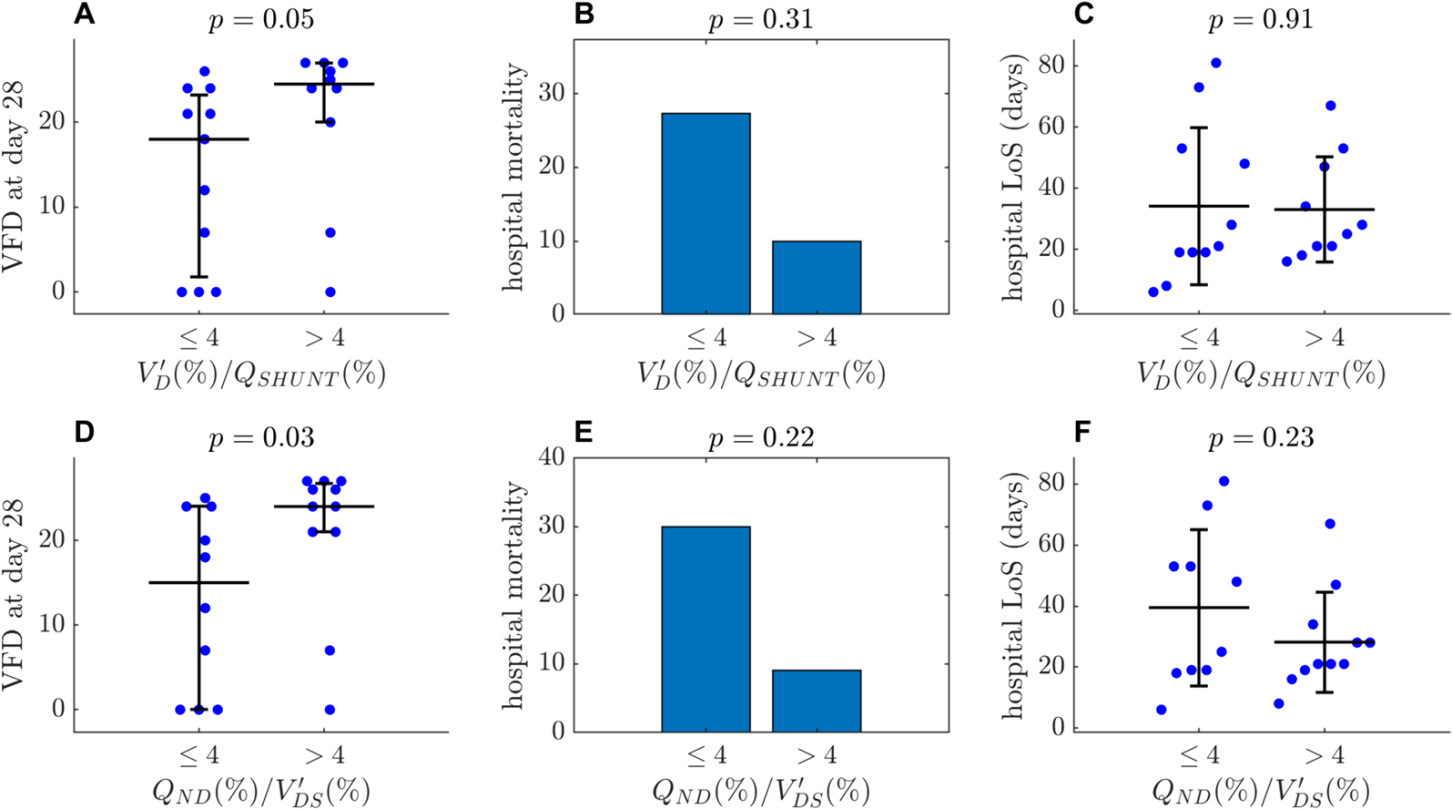
Physiological compensation and outcomes. Patients are divided into two groups according to the median values of our indices of shunt and dead space compensation (could be rounded to 4 in both cases). Uneven bars represent the interquartile range of non-normally distributed data, while equal bars represent standard deviation of normally distributed data

Patients with less effective shunt compensation, below the median value ( $V_{D}^{\prime } /Q_{SHUNT} < 4$) had fewer ventilator-free days (VFDs) at day 28 (18 (1.75–21.25) vs. 24.5 (20–27); *p* = 0.05) and a trend towards higher hospital mortality (3/11 (27%) vs. 1/10 (10%); *p* = 0.31) (Fig. 7A–C).

Similarly, patients with lower capacity for dead space compensation ( $Q_{ND} /V_{DS}^{\prime } < 4$) had fewer VFD (15 (0–24) vs. 24 (21–27); *p* = 0.03) and higher hospital mortality (3/10 (30%) vs. 1/11 (9%); *p* = 0.22) and also tended to spend more days in the ICU (18 (11–41) vs. 7 (3–15); *p* = 0.07) and in the hospital (40 ± 26 vs. 28 ± 16; *p* = 0.23) (Fig. 7D–F).

## Discussion

This study described a novel method which, through EIT-based ventilation and pulsatility data, corrects relative V′/Q maps to obtain meaningful non-invasive absolute maps of V′/Q mismatch in patients with ARDS. Validation with reference V′/Q maps calibrated with invasive monitoring showed close correspondence. We also proposed two indices derived from such maps, correlated with the intensity of shunt and dead space compensation [1, 24], and found that shunt compensation improves oxygenation, and that dead space compensation seems to be influenced by airway pressure. Finally, explorative analyses indicated a correlation between less effective compensation of shunt and dead space and worse clinical outcomes.

The ability of EIT to image ventilation is well established [9]. More recently, interest has increased in its capability to assess lung perfusion [27, 28], in particular with indicator-based methods, most commonly hypertonic saline [29]. Lung perfusion by EIT has been validated in porcine models against contrast-enhanced computed tomography (CT) [29–31] and nuclear imaging [10, 32, 33] and was found to approximate closely the observed changes in perfusion CT scans induced by the progression of lung injury and by PEEP changes in a swine model of ARDS [31]. Further clinical and experimental research work is needed to translate these experimental findings into clinical practice. Commercially available EIT devices may already be used for this purpose, albeit requiring additional offline analysis with customized software in most cases [28].

Ventilation/perfusion mismatch lies at the basis of most pathophysiologic derangements in acute respiratory distress syndrome. It would be crucial that functional EIT maps of ventilation and perfusion could be superimposed to generate accurate pixel-level V′/Q ratios for proper use and interpretation. This approach has been validated both in animal and human studies. Hentze et al. assessed the response to changes in PEEP of V′/Q maps in pigs [34], but then refrained from showing new maps in their later works [32, 35], as they felt they could not ensure accurate spatial matching due to a dorsal shift in ventilation and a ventral shift in perfusion, as compared to single-photon emission computed tomography (SPECT). Larrabee and colleagues [36] altered V′/Q by changing cardiac output either pharmacologically or with controlled hemorrhage and found good agreement between EIT and standard metrics of global shunt [7] and dead space [1, 37]. In line with Hentze et al., they found their EIT images to be shifted, nonetheless they could demonstrate good trending ability. Meanwhile, clinical studies in ARDS patients assessed V′/Q mismatch by EIT in response to PEEP [22, 38, 39], positional therapy [39–42] and pulmonary vasodilation with nitric oxide [43], finding moderate to weak correlations with known bedside indices of V′/Q mismatch [42–44]. It must be noted that a difference between imaging techniques and functional indices is to be expected [45]. While the correspondence between V′ and Q maps could be better characterized, the potential of V′/Q at EIT to yield potentially relevant information at the bedside has been highlighted also by the finding that worse mismatch predicts unfavorable outcomes in ARDS [44]. Taken together, this evidence demonstrates the feasibility and suggests the physiological soundness and potential clinical relevance of deriving V′/Q maps from EIT.

Unfortunately, a major hindrance to the applicability of the technique is the need for invasive monitoring for calibration of perfusion. Indeed, with current technology, EIT can only provide information about the relative spatial distribution of ventilation and perfusion [46], which has to be multiplied by minute volume (MV), easily obtained by the ventilator spirometer in intubated patients, and cardiac output (CO), which needs invasive and seldomly used monitoring. While accurate and precise CO estimates can be obtained noninvasively by transthoracic echocardiography [47], this technique requires trained operators [48]. Without this calibration, EIT is only able to track V′/Q changes over time. Indeed, the ratio between MV and CO may significantly affect results. In particular, as in critical patients minute volume is normally higher than cardiac output, relative EIT measurements were found to give unprecise estimates of V′/Q compartments [11]. Here, we confirmed that relative V′/Q maps obtained by sheer superimposition of relative ventilation and perfusion images underestimate the high V′/Q compartment (V′/Q ranging from 1.25 to 10) and overestimate the low V′/Q compartment (V′/Q ranging from 0.1 to 0.8). We also found that, as one may expect, relative maps result in reduced variability of V′ and Q distributions over V′/Q ratios, as they fail to capture the variability due to differing MV/CO ratios between patients. Once translated into clinical practice, this may lead to unnecessary interventions, and it undermines the possibility to compare results between patients and within the same patient if either haemodynamics or ventilatory settings change.

Precise minute ventilation may be unavailable during non-invasive ventilation and, more importantly, despite the decreasing invasiveness of available devices [49], cardiac output monitoring is currently reserved to hemodynamically unstable patients, who are a minority of ARDS patients. To circumvent this limitation, we built on previous evidence demonstrating the correlation between stroke volume and the cardiac-related pulsatility signal superimposed on EIT ventilation traces [12, 13]. While the accuracy of EIT does not yet meet the required specifications for hemodynamic monitoring in the critically ill [12, 50], in our cohort it was sufficient to correct the bias of relative V′/Q ratios without using information from invasive monitoring. By directly estimating the MV/CO ratio, our method theoretically eliminates the need to measure both, thus considerably extending the applicability of V′/Q matching by EIT to otherwise minimally monitored patients.

Novel indices that merge corrected ventilation and perfusion information could also be developed to be robust against changes in minute volume and cardiac output. Some features unaffected by MV and CO, such as unmatched units have already proven to be able to follow pathophysiological changes at the bedside [43] and to predict outcomes [44]. Here, we found that EIT calibration mostly affects the high and low V′/Q compartments, while ventilation and perfusion to very low (< 0.1) and very high (> 10) V′/Q ratios, which we defined as shunt and dead space [22], are almost unchanged. This is because they are influenced mostly by units with complete absence of ventilation and perfusion. In an explorative analysis, we developed two indices ideally representing physiological compensation mechanisms: hypoxic vasoconstriction and hypocapnic bronchoconstriction, which limit the consequences of shunt and dead space, respectively [1]. We observed that higher $V_{D}^{\prime } /Q_{SHUNT}$ values, suggesting either less severe disease, or more efficient hypoxic vasoconstriction, correlate with improved shunt fraction and P/F ratio. Less prone to interpretation is the finding that higher $Q_{ND} /V_{DS}^{\prime }$, theoretically suggesting compensatory redistribution of ventilation, correlates with lower plateau and end-expiratory pressures. This could be due partly to milder disease and partly to the diminished need to correct ventilation distribution at lower airway pressure, as overdistension and non-dependent hypoperfusion are less likely to occur. Of note, both indices are almost unaffected by V′/Q map calibration, as their numerators are, by definition, independent from it, while we found their denominators to be quite independent from MV/CO changes.

The concordant trends in predicting lower ventilator-free days, and higher hospital mortality and hospital length of stay, generate the hypothesis that these two indices mirror severity of lung injury, with loss of physiological mechanisms of regional compensation.

Our study has several limitations. Above all, the strength of correlation between our proposed non-invasive calibration procedure and the invasive gold standard is moderate. However, our work should be viewed as a proof-of-concept study, proving feasibility and the absence of major biases. Technical and algorithmic improvements will likely increase the strength of correlation. In particular, we employed the method by Deibele et al. [20] to separate cardiac-related oscillations. ECG-gated EIT would probably have been more suitable, especially for noisy data. Moreover, our method for defining pulmonary and cardiac regions of interests (ROI) for pulsatility is rather simple and might have excluded some collapsed lung regions. Although the pulsatility signal in the atelectatic lung may be especially prone to bias [10], the possible dependence of our calibration factor on mechanical ventilation settings, especially PEEP, could not be sufficiently addressed with our dataset. ECG-gating would have provided a better reference for pulsatility phase imaging, potentially allowing more accurate and ventilation-independent ROI definition [48, 51]. The second major limitation of our study is the absence of a validation cohort to assess our regression model for calibration. This study provides clues about the intrinsic coherence of our method, so that further research is warranted. As we did not employ volume capnography, we lacked an adequate reference for dead space assessment and could not correct minute volume with anatomical dead space to obtain alveolar ventilation [36]. We chose not to employ a fixed proportion for anatomic dead space, as previous authors did [11, 39], since most of the strength of EIT-based V′/Q lies in its trending ability [36], while central compression of EIT reconstructions [52] and positional bias [32] devoid of interest the comparison with reference ranges for shunt and dead space fractions. Finally, we do not normally observe major hemodynamic derangements during the 20 s breath hold required for measuring lung perfusion at EIT, but cardiac output measurements could not be simultaneous due to technical reasons (thermodilution bolus is known to affect impedance [53]) and hence we cannot exclude an influence.

## Conclusions

We described a calibration method for calculating corrected regional V′/Q mismatch that closely corresponds to the absolute one obtained with use of invasive hemodynamic monitoring. We proposed two indices of physiological compensation of shunt and dead space derived from V′/Q maps and described interesting physiological interactions of these indices with oxygenation and ventilation settings, and with clinical outcomes. Although further research is needed, our proposed method constitutes a step forward towards the utilization of bedside V′/Q imaging by EIT as a valuable non-invasive bedside measure of the severity of ARDS and, possibly, as a guide for personalized treatment, even in early less monitored phases of the disease. For example, the impact on V/Q mismatch of interventions known to impact cardiac output (e.g., PEEP increase) could be more precisely evaluated by our novel experimental approach”.

### Supplementary Information

Additional file 1 (DOCX 237 KB)

## Data Availability

The datasets used and/or analysed during the current study are available from the corresponding author on reasonable request.

## Abbreviations

ARDS: Acute respiratory distress syndrome
CO: Cardiac output
EIT: Electrical impedance tomography
fEIT: Functional EIT image
FEM: Finite element model
HR: Heart rate
ICU: Intensive care unit
K_C_: Calibration factor
MV: Minute ventilation
Q: Perfusion
$Q_{ND} /V_{DS}^{\prime }$: Ratio between perfusion to non-dependent units and ventilation to dead space
RR: Respiratory rate
SAPS: Simplified acute physiology score
SOFA: Sequential organ failure assessment
SV: Stroke volume
V′: Ventilation
$V_{D}^{\prime } /Q_{SHUNT}$: Ratio between ventilation to dependent units and perfusion to shunted units
V′/Q: Ventilation/perfusion ratio
V′/Q-ABS: Absolute (invasive) V′/Q (see text)
V′/Q-CORR: V′/Q calibrated with EIT data (noninvasive)
V′/Q-REL: Relative (uncalibrated) V′/Q (see text)
VFD: Ventilator-free days
∆Z: Impedance change
∆Z_CR_: Cardiac-related impedance change
∆Z_TIDAL_: Impedance change due to tidal ventilation
