# Supplementary material for: Absolute values of regional ventilation-perfusion mismatch in patients with ARDS monitored by electrical impedance tomography and the role of dead space and shunt compensation
Source: Crit Care. 2024 Jul 15;28:241. doi: 10.1186/s13054-024-05033-8 (PMC11251389; doi:10.1186/s13054-024-05033-8)
Supplement: Supplementary file 1 — Additional file 1 (DOCX 237 KB) [file 13054_2024_5033_MOESM1_ESM.docx]

**Supplementary Materials**

**Methods**

Signal Pre-processing

Pre-processing differed according to the type of functional EIT map:

- Ventilation maps: no low-pass filtering was applied, traces were visually inspected to ensure accurate tidal detection.
- Pulsatility maps: the band-pass FIR filter within the referenced algorithm (1) was adjusted, as suggested by the authors: a minimal filter order design was applied to ensure a filter transition band between the second harmonic of ventilation and heart rate.
- Perfusion maps: data were low-pass filtered at 0,83 Hz before perfusion analysis.

ROI Definition for Pulsatility Analysis

First, we identified a cardiac region of interest (ROI) by calculating the phase of pulsatility for each pixel relative to the sum of all pixels, as previously described (2). We then identified a lung ROI by subtracting the cardiac ROI to the ROI including ventilated pixels (regional ventilation >20% of the maximum within the ventilation map). We summed all pixels within the cardiac and the lung ROI, thus obtaining two cardiac-related impedance changes, respectively:

- $\Delta Z_{CR,lung}= \sum_{lung ROI} {\Delta Z}_{CR,PX}$ (1)
- $\Delta Z_{CR,heart}= \sum_{heart ROI} {\Delta Z}_{CR,PX}$ (2)

In line with previous results (3), we found that $\Delta Z_{CR,lung}$ correlated better with stroke volume: we referred to it as $\Delta Z_{CR}$ in the paper and from this point forward in this Supplement.

**Results**

Calibration Factor and Ventilatory Settings

The calibration factor did not appear to correlate with PEEP (r=-0.12; p=0.613). If added to the linear model used to predict the MV/CO ratio based on the ratio of impedance changes (∆Z_TIDAL_*RR/∆Z_CR_*HR), no significant effect PEEP was found, leaving the coefficient estimates almost unchanged (see below). When added to the model, the interaction term was non-significant as well (p=0.981, estimates not shown due to overfitting).

|  | estimate | p-value |
| --- | --- | --- |
| Intercept | 0.41±0.42 | 0.349 |
| ∆Z_TIDAL_*RR/∆Z_CR_*HR | 0.52±0.15 | 0.002 |
| PEEP (cmH2O) | 0±0.03 | 0.926 |

Calibration Factor and Cardiac Output

A cardiac output estimate can be derived from the calibration factor by using the minute volume measurement: CO≈MV/Kc. The relative error (±46%) for this measurement is presented in the Bland-Altman plot in Figure S1. This figure is outside the usual 30% range suggested for CO monitors, but close to the 45% figure proposed for minimally invasive devices (4). It must be noted that this estimate suffers both from the intrinsic error of EIT and from the inaccuracy of minute volume measurement (MV) at the ventilator, this is why the limits of agreement with the MV/CO ratio are presented instead in the main paper.

Figures

Fig. S1


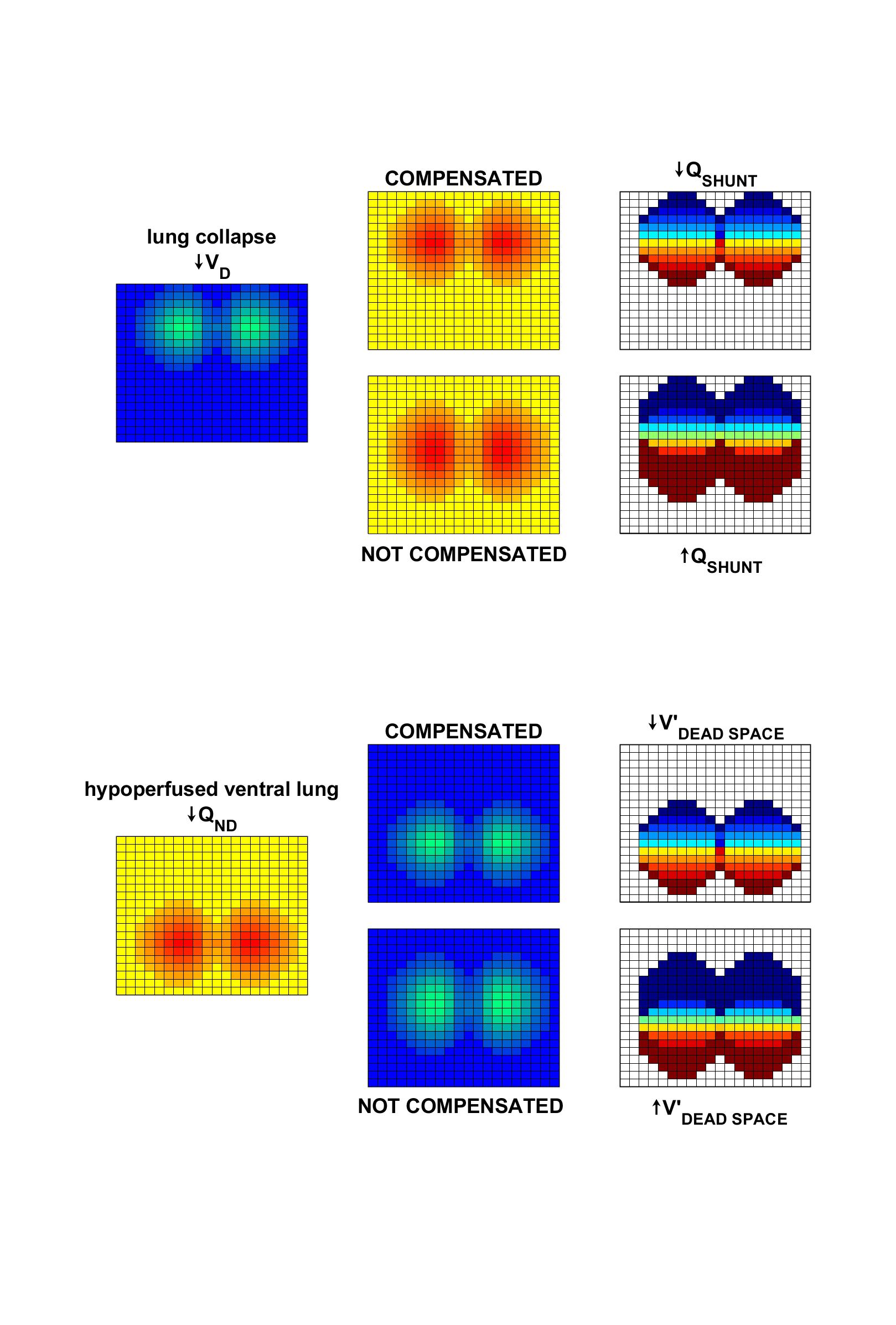


Schematic for the indices of physiologic compensation. In the upper panel, a decrease in dorsal ventilation (V’_D_) due to lung collapse is portrayed: ideally, if hypoxic vasoconstriction ensues perfusion will be redistributed ventrally, thus reducing Q_SHUNT_ and increasing the V’_D_/ Q_SHUNT_ ratio. In the bottom panel, a decrease in ventral perfusion (Q_ND_), possibly due to increased airway pressure, is displayed: ideally, if hypocapnic pneumoconstriction ensues ventilation will be redistributed dorsally, thus reducing V’_DEAD SPACE_ and increasing the Q_ND_/ V’_DEAD SPACE_ ratio.

Fig. S2


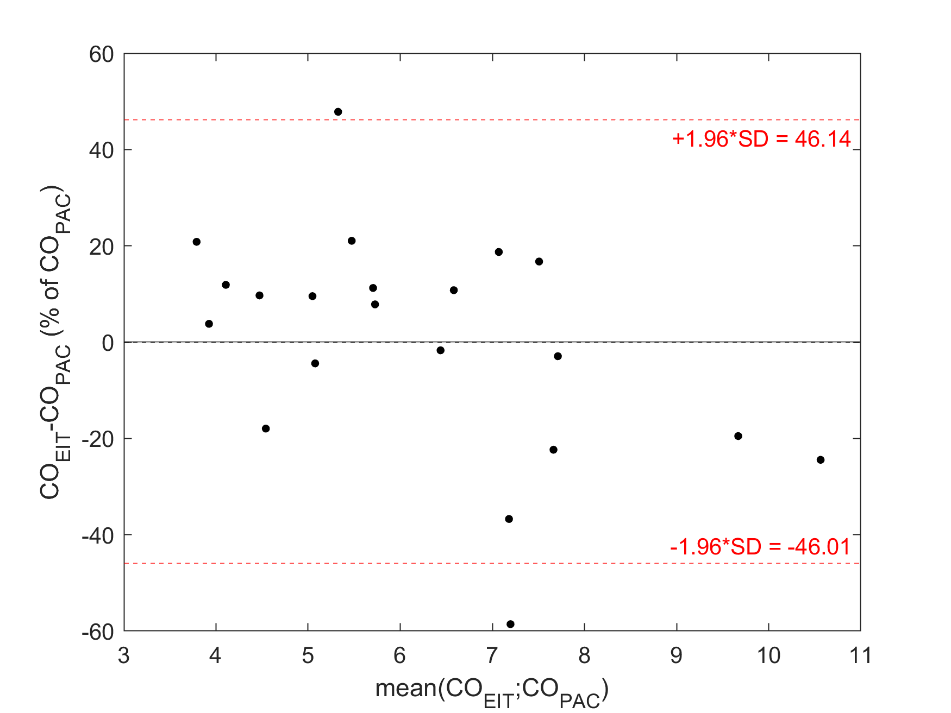


Bland-Altman plot for the agreement between the clinical gold standard for cardiac output, that is thermodilution via a pulmonary artery catheter (PAC) and cardiac output (CO) derived from EIT and minute volume measurements from the ventilator spirometer. CO_PAC_ is CO measured by the PAC. CO_EIT_ is the CO derived from minute volume and the proposed calibration factor.

Fig.S3


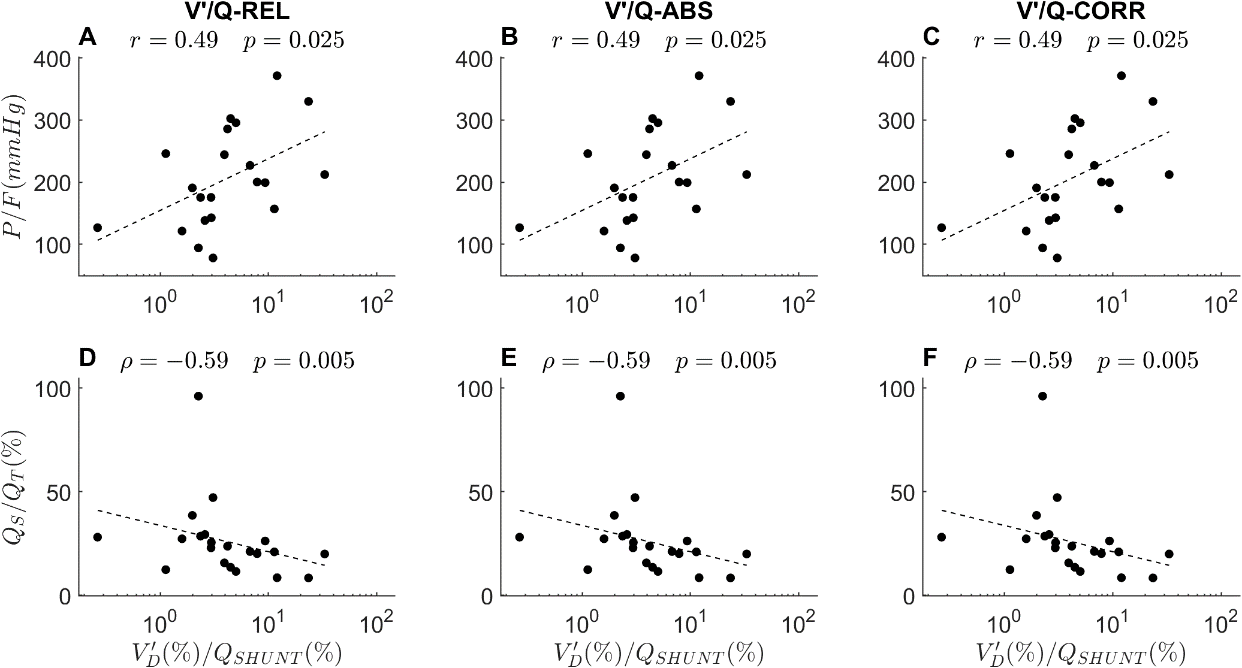


Shunt compensation and classical bedside measures of severity and shunt. See text for abbreviations. Indices from V’/Q maps left uncorrected (V'/Q-REL, *left column*), corrected with invasive measurements (V’/Q-ABS, *middle column*) and with our proposed calibration factor (V’/Q-CORR) are plotted against (A-C) P/F and (D-F) shunt fraction (Q_S_/Q_T_) from blood gases.

Fig.S4

**
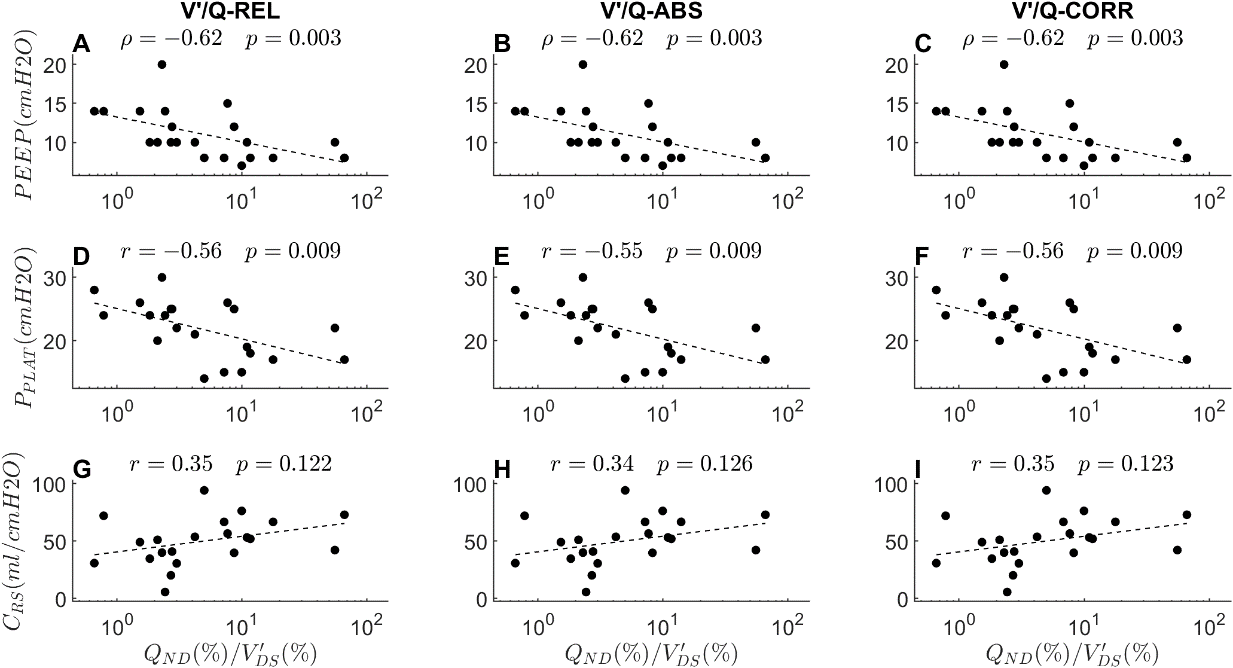
**

Dead space compensation and respiratory system mechanics. See text for abbreviations. Indices from V’/Q maps left uncorrected (V’/Q-REL, *left column*), corrected with invasive measurements (V’/Q-ABS, *middle column*) and with our proposed calibration factor (V’/Q-CORR, *right column*) are plotted against (A-C) positive end expiratory pressure (PEEP), (D-F) plateau pressure (P_PLAT_) and (G-I) respiratory system compliance (C_RS_).

**References**1. Deibele JM, Luepschen H, Leonhardt S. Dynamic separation of pulmonary and cardiac changes in electrical impedance tomography. Physiol Meas. 2008 Jun 1;29(6).

2. Frerichs I, Pulletz S, Elke G, Reifferscheid F, Schädler D, Scholz J, et al. Assessment of Changes in Distribution of Lung Perfusion by Electrical Impedance Tomography. Respiration. 2009;77(3):282–91.

3. Braun F, Proença M, Adler A, Riedel T, Thiran JP, Solà J. Accuracy and reliability of noninvasive stroke volume monitoring via ECG-gated 3D electrical impedance tomography in healthy volunteers. PLoS ONE. 2018 Jan 1;13(1).

4. Peyton PJ, Chong SW. Minimally Invasive Measurement of Cardiac Output during Surgery and Critical Care. Anesthesiology. 2010 Nov 1;113(5):1220–35.
